# Supplementary material for: iGenSig-Rx: an integral genomic signature based white-box tool for modeling cancer therapeutic responses using multi-omics data
Source: BMC Bioinformatics. 2024 Jun 19;25:220. doi: 10.1186/s12859-024-05835-1 (PMC11186173; doi:10.1186/s12859-024-05835-1)
Supplement: Supplementary file 1 — Additional file 1. Additional description for retrieval of breast cancer clinical trial datasets and extracting multi-omics features from the datasets. [file 12859_2024_5835_MOESM1_ESM.docx]

**Additional file 1: Methods**

**Retrieval of breast cancer clinical trial datasets**

We profiled patient clinical data, drug response data, and genomic data of breast cancer clinical trials focusing on HER2-positive patients treated by Trastuzumab. CALGB 40601 (ClinicalTrials.gov ID, NCT00770809; Registry identifier, NCI-2009-01073; Study registration date, 2008-10-09; dbGaP Accession Number, phs001570.v2.p1, ver.74825-8) and ACOSOG Z1041 (ClinicalTrials.gov ID, NCT00513292 and NCT00353483; Registry identifier, NCI-2009-00341; Study registration date, 2007-08-06; dbGaP Accession Number, phs001291.v1.p1, ver.69443-7) study datasets were retrieved from NCBI dbGaP. NOAH (NeOAdjuvant Herceptin; ClinicalTrials.gov ID, NCT04538079; Study registration date, 2020-03-05) [1] data were retrieved from NCBI GEO (GSE22226). NSABP B-41 (ClinicalTrials.gov ID, NCT01850628; Study registration dates, 2013-05-01) data were obtained from Georgetown University through Dr. Gong Tang. For iGenSig-Rx modeling, we included only HER2-positive and Trastuzumab-treated patients and excluded patients who failed RNA sequencing. These included only THL (T, Paclitaxel; H, Trastuzumab; L, Lapatinib; n=108) and TH (n=109) arms but excluded TL (n=60) arm in CALGB 40601. All patients in ACOSOG Z1041 (n=48) are HER2-positive and Trastuzumab treated. In NOAH, only arm3 (n=63) patients of HER2-positive and Trastuzumab-treated were included **(Table 1)**.

**Extracting multi-omics features from clinical trial datasets**

CALGB 40601 (n=265) and ACOSOG Z1041 (*n*=42) studies provide RNA-seq raw data files with Illumina reads. We used Rsubread (v. 2.4.2) [2] to align sequence reads to reference genome and used edgeR (v. 3.32.0) [3] and Limma (v. 3.46.0) [4] R packages (Bioconductor release 3.8) to normalize gene expression level to transcripts per million (TPM) [5]. We aligned the sequence read to the GRCh38 human genome reference sequence obtained from the Gencode database (<https://www.gencodegenes.org/human/>) and mapped the aligned sequences to Ensembl gene IDs Gencode v29). In addition, we performed STAR-Fusion on the RNA-seq data and identified chimeric transcripts based on the junction and spanning reads which are mapped to the reference genome [6].

CALGB 40601 study provides whole-exome sequencing (WXS) data for tumor patients (*n*=218) and matched peripheral blood mononuclear cells (PBMC) (*n*=198). We used GATK Mutect2 (GATK v. 4.1.2.0) to detect somatic variants with high sensitivity and specificity by comparing both tumor and normal samples [7]. Sequence mutations identified by Mutect2 were annotated with a gene symbols, mutation types, and predicted protein alterations using ANNOVAR [8]. Likewise, we obtained somatic mutation data of ACOSOG Z1041 (*n*=48), which were identified by GATK Mutect2, from the supplementary table of the reference paper [9].

NOAH expression data are generated by Affymetrix microarray U133 Plus 2.0. We used Single Channel Array Normalization (SCAN, v. 2.32.0) R package and custom Chip Description Files (CDF) provided by the Brainarray database (<http://brainarray.mbni.med.umich.edu>) [10] to normalize the data and map microarray probes to Ensembl gene IDs. As NOAH does not provide the patients’ WXS or somatic mutation data, we used only gene expression data.

Next, we extracted the gene expression, somatic mutations, and chimeric transcripts datasets and generated an integrated genomic feature file. For gene expression data, we eliminated zero gene expression values by adding half of the minimum expression value across patients. Then, we calculated log2 transformed fold changes of the expression values by comparing them to the trimmed mean of expression values (excluding the 10% largest and 10% smallest values). Based on the mean and standard deviation (SD) of fold changes, we assigned the genomic features into the following overlapping groups: ‘Up_Level1’ group with the fold change above Mean+1*SD for a given gene; ‘Up_Level2’ group with the fold change a Mean+2*SD; ‘Up_Level3’ … ‘Up_Level5’ as follows; and ‘Up_Level6’ group with the fold change above Mean+6*SD. Likewise, ‘Down_Level1’, ‘Down_Level2’, … and ‘Down_Level6’ grouped patients based on Mean-1*SD, Mean-2*SD, … Mean-6*SD. The 12 ‘Levels’ were labeled as genotypic features for each given gene, and the binary genomic features were compiled in a Genomic Matrix Transposed (GMT) file format. Genomic features of the gene expression fold change in between mean+/- 1*SD are generally not considered as overexpressed or underexpressed genes thus are excluded from the features. Similarly, we extracted binary genomic features to represent point mutations. The nonsynonymous somatic mutations such as missense, nonsense, and frameshift are assigned as mutation features, and each recurrently mutated gene was assigned as a separate feature. To prevent bias, we excluded the genomic features defining fewer than ten subjects.

**Determining the D-line for calculating iGenSig-Rx scores**

Based on therapeutic sensitive or resistant patients in CALGB 40601, we calculated each patient’s treatment sensitive or resistant iGenSig-Rx scores **(Figure 1)**. We then sorted all patients based on the ratio of sensitive and resistant iGenSig-Rx scores in descending order. Scanning each patient from the top to the bottom of this sorted patient list, the percentage of sensitive patients ranked higher than each patient is calculated as:

6) ${Percentage}_{i}|sensitive=\frac{\sum_{j=1}^{i} N_{j}(N_{j}=1 if {patient}_{j}\in drug sensitive set; N_{j}=0 if {patient}_{j}\notin sensitive)}{Total number of sensitive patients}$

Similarly, the percentage of resistant patients ranked higher than each patient is calculated as:

7) ${Percentage}_{i}|resistant=\frac{\sum_{j=1}^{i} N_{j}(N_{j}=1 if {patient}_{j}\in drug resistant set; N_{j}=0 if {patient}_{j}\notin resistant)}{Total number of resistant patients}$

Then the Youden Index for determining the optimal ratio to separate sensitive from resistant patients was calculated as below:

8) $Youden Index=max({percentage}_{i}\left| sensitive-{percentage}_{i} \right|resistant)$

In a two-dimensional plot by the i*GenSig-Rx.sensitive* and *iGenSig-Rx .resistant* scores, patients will be placed according to the *iGenSig-Rx.sensitive* and *iGenSig-Rx.resistant* scores. The slope of the dividing line (D-line) for sensitive and resistant patients is determined by Youden Index.

**Benchmark the performance of the iGenSig-Rx models and noise simulations**

We randomly selected 90 % of CALGB 40601 patients as the training set and assigned the rest of 10% subjects as the internal test set and performed this randomized sampling ten times. ACOSOG Z1041 and NOAH datasets were used as external validation set of our predictive model to assess the applicability to an independent dataset. First, we investigated whether iGenSig-Rx scores are associated with other clinical subtypes, such as treatment arms, menopausal status, and tumor stages. Then, we examined the correlation between the expression of HER2, ER, and PR vs iGenSig-Rx scores. To compare the predictive powers between HER2, ER, and PR expression and the iGenSig-Rx model, we used the gene expression of HER2, ER, and PR as predictive variables and calculated predictive performance AUROC in ACOSOG Z1041 and NOAH.

Sometimes, sequencing bias or misreading of genomic data can make errors in genomic features. To simulate the common biases in genomic features resulting from experimental variations or sequencing errors, we generated false-positive or false-negative genomic features in either CALGB 40601 or ACOSOG Z1041. By randomly deleting or inserting genomic features, we generated simulated genomic feature sets with 5, 10, 15, 20, or 25% error rates. We repeated the simulations five times for each error rate and applied them to the ten random sampling of CALGB 40601 trainsets or ACOSOG Z1041 validation sets. We then applied the same sets of genomic features with or without error simulations to iGenSig-Rx modeling or the deep learning and machine learning modeling methods to compare the resilience of the iGenSig-Rx modeling with the AI-based methods. In the next section, AI-based approaches that use Autoencoder for dimensional reduction and machine learning prediction algorithms are explained in detail.

**The resilience of the iGenSig-Rx model against data devoid of drug target and hormone receptor genes**

As the gene expression of HER2, ER, and PR are associated with the iGenSig-Rx model, we examined the resilience of the iGenSig-Rx model on Trastuzumab-treated breast cancer patients devoid of a drug target and hormone receptor genomic feature. We removed the Trastuzumab target gene feature, HER2, hormone receptors, ER, and PR genomic features from CALGB 40601, ACOSOG Z1041, NOAH, or NSABP B-41 assessed its performance on the internal test and the external validation sets. We performed the exact ten random sampling to train the model with CALGB 40601 90% data and test with CALGB 40601 10% data and validate it on ACOSOG Z1041, NOAH, and NSABP B-41 data. This is to test if the iGenSig-Rx predictions rely on the genomic features of the primary drug targets and hormone receptor gene expressions.

**Deep learning autoencoder and machine learning prediction based on genomic features**

Previous studies demonstrated the high predictive power of combined multi-omics data with deep learning and machine learning approaches in drug response prediction [11, 12]. Following the studies, we applied the deep learning method autoencoder to perform unsupervised representation learning for dimensionality reduction and machine learning prediction algorithms for supervised learning of therapeutic responses using the low dimensional features generated by autoencoder and compared their prediction performances with our iGenSig-Rx method. The Autoencoder model was developed using the genome-wide gene expression and mutation features that we compiled. We used the same training and external validation sets of breast cancer clinical trial data as in iGenSig-Rx modeling. We used keras R package (ver.2.13.0, which provides a high-level interface for building and training neural networks. With keras, we customized layers, loss functions, and hyperparameters. In this study, we employed an Autoencoder for dimension reduction of the input data, not for prediction. In the dimension reduction by Autoencoder, we didn’t get any overfitting issue. To monitor the Autoencoder performance, we fine-tuned the embedding layer size, unit sizes, dropout rate, number of synthetic features, and training iterations using 10 permuted training sets for dimension reduction. We then applied elastic net for prediction on the external validation sets. We verified that changes in parameters did not affect the performance of Autoencoder dimension reduction and elastic net prediction (**Supplementary Figure 6**). Through the fine-tuning process, we constructed the Autoencoder model with three embedding layers, with unit sizes of 1300, 552, and 235 in each layer, using the "sigmoid" activation function. The encoded synthetic feature size was set to 100. We then applied the unsupervised representation of the genomic correlates to supervised learning methods, including elastic net, artificial neural network, Random Forest (RF), and support vector machine (SVM) for prediction modeling.

Elastic net is a regression method that combines lasso and ridge regularization with the two hyperparameters, alpha and lambda. Alpha is a mixing parameter to define the relative weight of the lasso and ridge penalization terms, and lambda determines the amount of shrinkage [13]. We identified alpha with the best tuning and optimized for predictive performance over a range of lambdas. Regression was performed using the glmnet R package (ver. 4.0.2). Artificial neural network is a non-linear learning model and contains hidden layers that use backpropagation to optimize input variables’ weights to improve the model’s predictive power. For artificial neural network prediction, we specified three hidden layers with 5, 3, and 10 nodes and a 0.01 threshold for the partial derivatives of the error function using the neural net R package (ver. 1.44.2). We implemented an RF regression model using the random forest R package (ver.4.6.14). We specified 1,000 trees to grow and ensure every object gets predicted multiple times. We used SVM with the linear kernel method, ‘svmLinear2’, provided by the caret R package (ver. 6.0.86). We specified tuneLength=10 in the tuning parameter grid and accuracy metric.

**CSEA analysis for interpreting the signature pathways underlying iGenSig-Rx models.**

To examine the characteristics of the integral genomic features for Trastuzumab-sensitive or resistant-breast cancer patients, we analyzed gene pathway signatures in the genomic features. The genes involved in the significant genomic features of the iGenSig-Rx model were extracted and classified into drug-sensitive or resistant contributing genes. The positive contributing genes were defined by upregulated genes from the drug-sensitive or resistant contributing genes **(Figure 5A)**. Down-regulated genes and nonsynonymous mutation genes are defined as negative contributing genes. The pathway enrichment in the positive or negative contributing genes for Trastuzumab sensitivity or resistance was analyzed by the Concept Signature Enrichment Analysis (CSEA) developed in our previous study **(Figure 5A)** [14]. The resulting top 30 pathways are disambiguated via correcting the crosstalk effects between pathways to reveal independent pathway modules [15]. A p-value < 0.01 is used as the cutoff for disambiguation. The functional associations between the significant pathways are then assessed using our CSEA method as we previously described [14]. The CSEA scores are scaled between -1 and 1 and visualized using a correlogram. The pathway network was visualized using the igraph R package (ver. 1.2.4.2).

**Statistical analysis**

Multiple logistic regression models are generated for pair-wise interactions of the iGenSig-Rx model with individual confounding clinical variables for interaction analysis. The multiple models are compared with the simple logistic regression model via the Chi-Square test implemented in the ‘ANOVA’ function. Paired or unpaired Student t-tests analyzed P-values of the box or violin plots. Two-sided tests are used when both directions are tested, such as when comparing the different modeling methods. One-sided tests are used to determine if there is a difference between groups in a specific direction, such as that defined by the iGenSig-Rx scores.

**References**

1. Gianni L, Eiermann W, Semiglazov V, Manikhas A, Lluch A, Tjulandin S, Zambetti M, Vazquez F, Byakhow M, Lichinitser M: **Neoadjuvant chemotherapy with trastuzumab followed by adjuvant trastuzumab versus neoadjuvant chemotherapy alone, in patients with HER2-positive locally advanced breast cancer (the NOAH trial): a randomised controlled superiority trial with a parallel HER2-negative cohort**. *The Lancet* 2010, **375**(9712):377-384.

2. Liao Y, Smyth GK, Shi W: **The Subread aligner: fast, accurate and scalable read mapping by seed-and-vote**. *Nucleic acids research* 2013, **41**(10):e108-e108.

3. McCarthy DJ, Chen Y, Smyth GK: **Differential expression analysis of multifactor RNA-Seq experiments with respect to biological variation**. *Nucleic acids research* 2012, **40**(10):4288-4297.

4. Ritchie ME, Phipson B, Wu D, Hu Y, Law CW, Shi W, Smyth GK: **limma powers differential expression analyses for RNA-sequencing and microarray studies**. *Nucleic acids research* 2015, **43**(7):e47-e47.

5. Wagner GP, Kin K, Lynch VJ: **Measurement of mRNA abundance using RNA-seq data: RPKM measure is inconsistent among samples**. *Theory in biosciences* 2012, **131**(4):281-285.

6. Haas BJ, Dobin A, Li B, Stransky N, Pochet N, Regev A: **Accuracy assessment of fusion transcript detection via read-mapping and de novo fusion transcript assembly-based methods**. *Genome biology* 2019, **20**(1):213.

7. Cibulskis K, Lawrence MS, Carter SL, Sivachenko A, Jaffe D, Sougnez C, Gabriel S, Meyerson M, Lander ES, Getz G: **Sensitive detection of somatic point mutations in impure and heterogeneous cancer samples**. *Nature biotechnology* 2013, **31**(3):213-219.

8. Wang K, Li M, Hakonarson H: **ANNOVAR: functional annotation of genetic variants from high-throughput sequencing data**. *Nucleic acids research* 2010, **38**(16):e164-e164.

9. Lesurf R, Griffith O, Griffith M, Hundal J, Trani L, Watson M, Aft R, Ellis M, Ota D, Suman VJ: **Genomic characterization of HER2-positive breast cancer and response to neoadjuvant trastuzumab and chemotherapy—results from the ACOSOG Z1041 (Alliance) trial**. *Annals of Oncology* 2017, **28**(5):1070-1077.

10. Dai M, Wang P, Boyd AD, Kostov G, Athey B, Jones EG, Bunney WE, Myers RM, Speed TP, Akil H: **Evolving gene/transcript definitions significantly alter the interpretation of GeneChip data**. *Nucleic acids research* 2005, **33**(20):e175-e175.

11. Ding MQ, Chen L, Cooper GF, Young JD, Lu X: **Precision oncology beyond targeted therapy: Combining omics data with machine learning matches the majority of cancer cells to effective therapeutics**. *Molecular Cancer Research* 2018, **16**(2):269-278.

12. Sharifi-Noghabi H, Zolotareva O, Collins CC, Ester M: **MOLI: multi-omics late integration with deep neural networks for drug response prediction**. *Bioinformatics* 2019, **35**(14):i501-i509.

13. Friedman J, Hastie T, Tibshirani R: **Regularization paths for generalized linear models via coordinate descent**. *Journal of statistical software* 2010, **33**(1):1.

14. Chi X, Sartor MA, Lee S, Anurag M, Patil S, Hall P, Wexler M, Wang X-S: **Universal concept signature analysis: genome-wide quantification of new biological and pathological functions of genes and pathways**. *Briefings in bioinformatics* 2020, **21**(5):1717-1732.

15. Donato M, Xu Z, Tomoiaga A, Granneman JG, MacKenzie RG, Bao R, Than NG, Westfall PH, Romero R, Draghici S: **Analysis and correction of crosstalk effects in pathway analysis**. *Genome research* 2013, **23**(11):1885-1893.
