# Supplementary material for: iGenSig-Rx: an integral genomic signature based white-box tool for modeling cancer therapeutic responses using multi-omics data
Source: BMC Bioinformatics. 2024 Jun 19;25:220. doi: 10.1186/s12859-024-05835-1 (PMC11186173; doi:10.1186/s12859-024-05835-1)
Supplement: Supplementary file 2 — Additional file 2. The principle and algorithm design of iGenSig-Rx modeling and correlation plots between iGenSig-Rx scores and hormone gene expression. [file 12859_2024_5835_MOESM2_ESM.pdf]

Additional file 2: Fig. 1

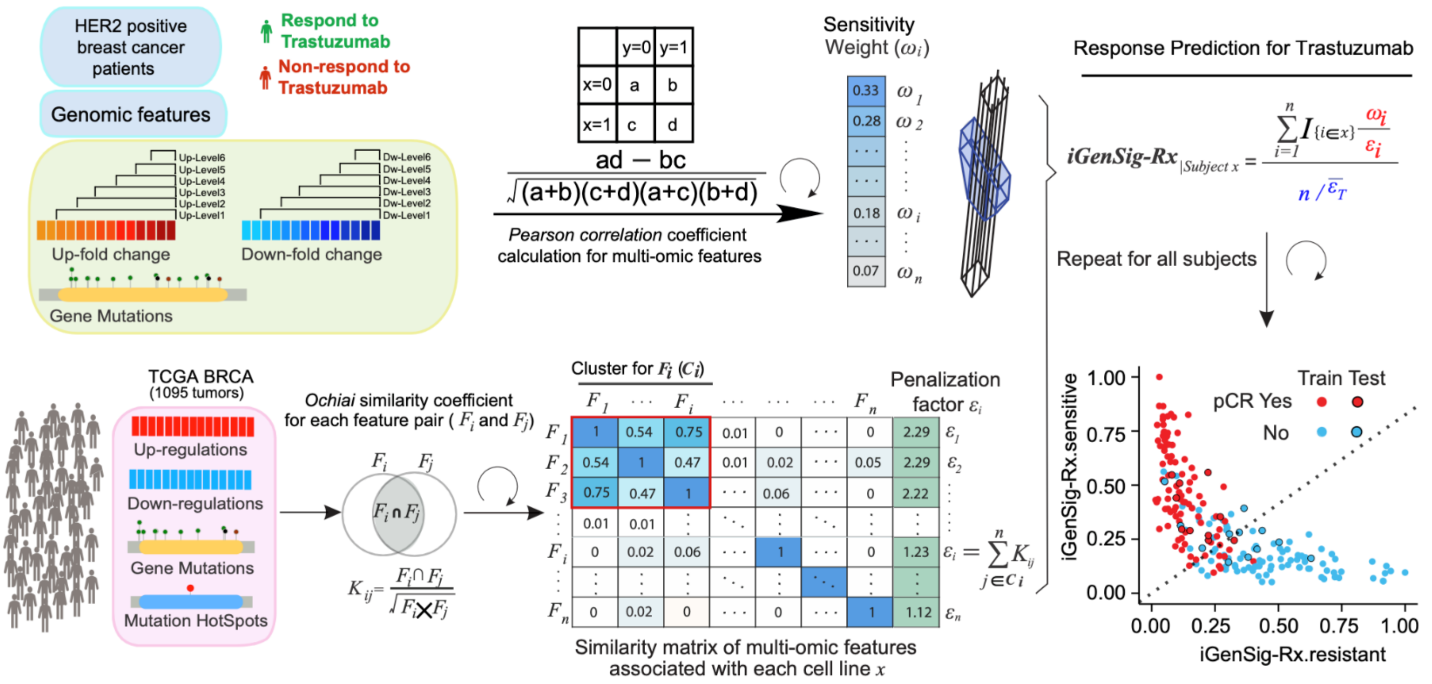

Additional file 2: Fig. 1. The principle and algorithm design of integral genomic signature analysis for predicting therapeutic response (iGenSig-Rx) in cancer. The upper panel shows the calculation of the weights for significant genomic features by using Pearson correlation. The weights represent the strength of association of genomic features with drug sensitive or resistant tumors and are used to calculate iGenSig-Rx sensitive or resistant scores of patient subjects. The lower panel shows the computation of a similarity matrix for genomic features based on TCGA Pan-Cancer dataset to penalize the redundancy between the genomic features associated with each patient x. The resulting penalization factors are formulated to calculate iGenSig-Rx sensitive or resistant scores, as depicted in the formula on the top right. In the dot plot of iGenSig-Rx sensitive and resistant scores for all patients, the best distinction line (D-line) for separating true sensitive from resistant patients is calculated. The distance of each patient to this D-line is defined as the "iGenSig-Rx" score. Positive and negative iGenSig-Rx values indicate sensitive and resistant response predictions, respectively.

Additional file 2: Fig. 2

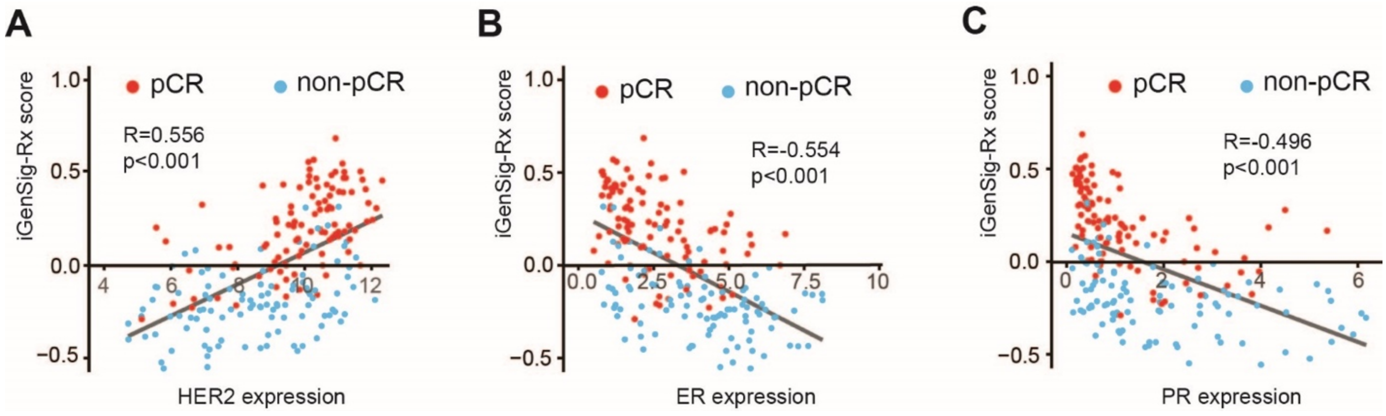

Additional file 2: Fig. 2. The correlation between iGenSig-Rx scores and HER2, ER, or PR expression. HER2 expression is positively, but ER and PR expressions are negatively correlate with iGenSig-Rx scores.

Additional file 2: Fig. 3

A. Up-regulated pathways in Trastuzumab sensitive BRCA patients

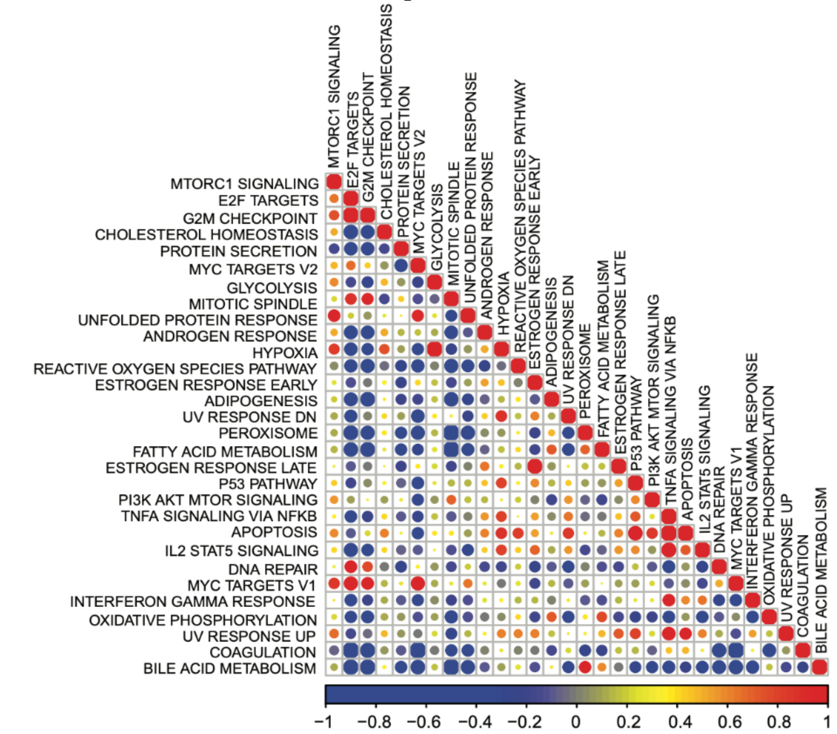

B. Up-regulated pathways in Trastuzumab resistant BRCA patients

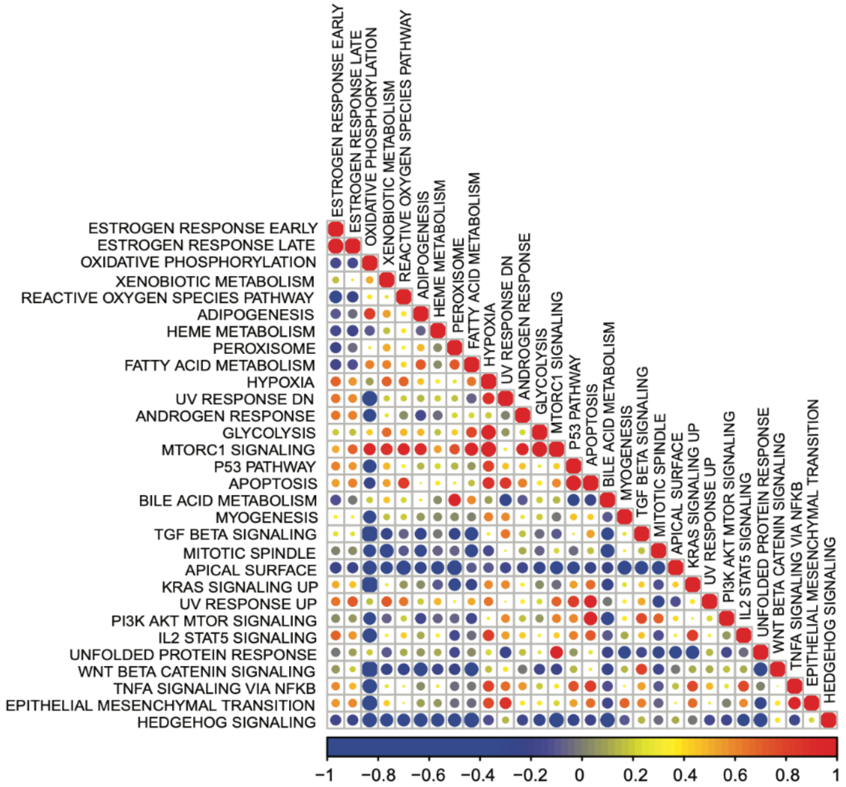

Additional file 2: Fig. 3. The upregulated pathway characteristics in the therapeutic (A) sensitive or (B) resistant iGenSig-Rx signature. The function associations between the significant pathways assessed by concept signature enrichment analysis (CSEA) are shown in red to blue color scales. The pathways are sorted in descending order based on their normalized enrichments scores; the most enriched pathways are ranked at the top.

Additional file 2: Fig. 4

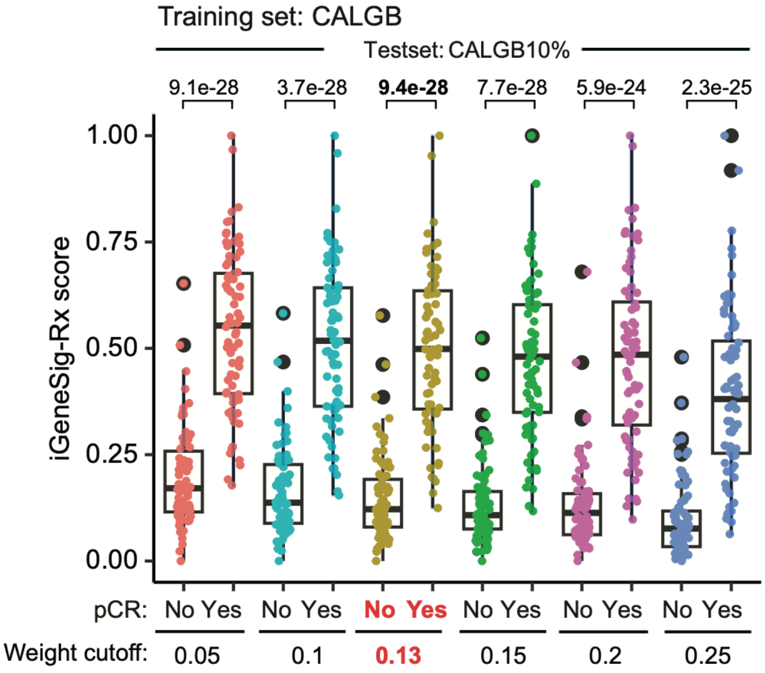

Additional file 2: Fig. 4. Testing the cutoffs for feature selection in iGenSig-Rx modeling. The iGenSig-Rx scores we benchmarked based on the training and testing sets of the CALGB dataset with various weight cutoffs. The weight cutoff 0.13, generated the most significant difference of iGenSig-Rx scores between non-pCR and pCR subjects in CALGB testing sets.

Additional file 2: Fig. 5

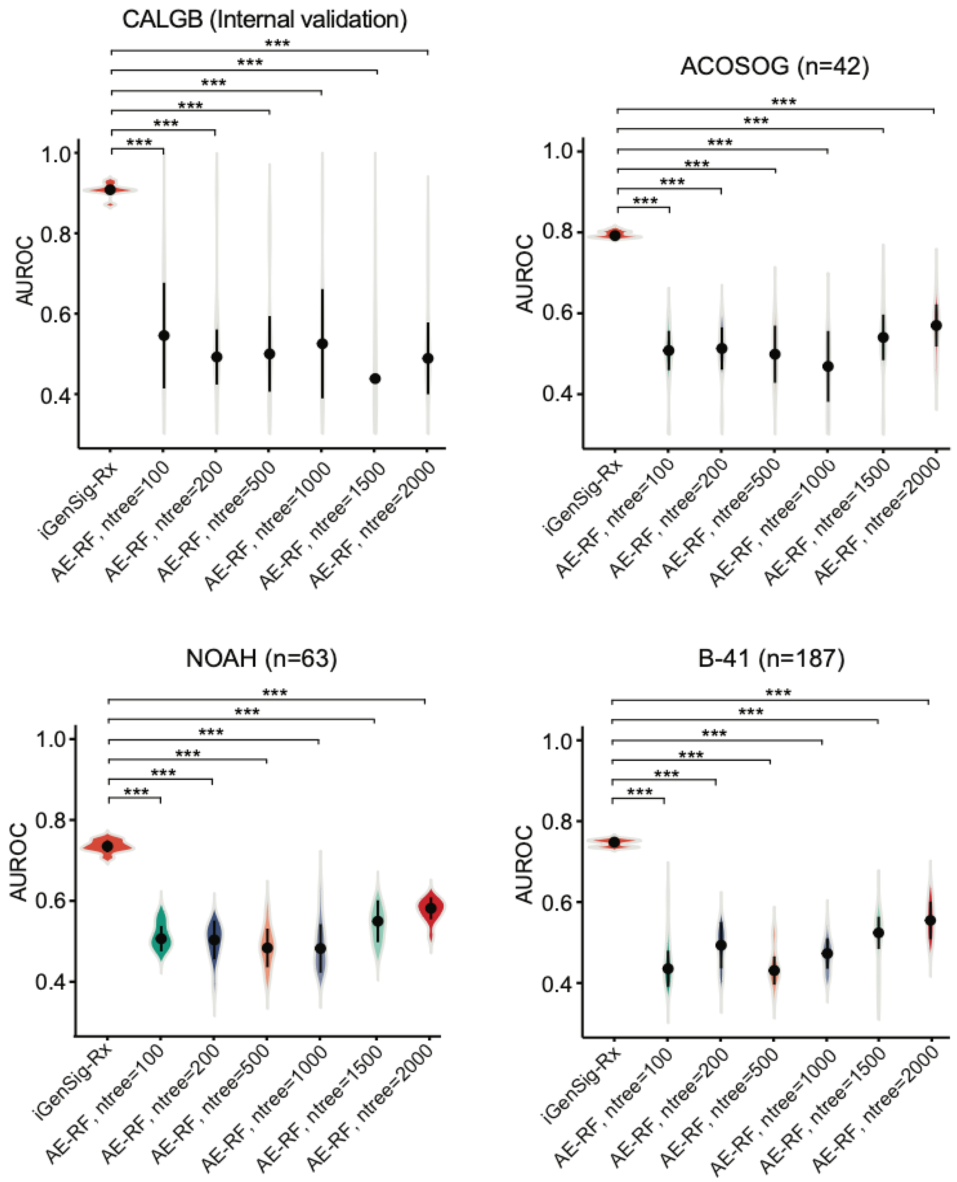

Additional file 2: Fig. 5. Predictive performance of autoencoder-random forest (AE-RF) with different tree depth. The number of trees at the range of from 100 to 2,000 was tested in AE-RF model. The prediction performance was not significantly affected by the different number of trees (no statistical difference) in the models. The AUROC of AE-RF models are all significantly lower than iGenSig-Rx. ***, p-value < 0.001.

Additional file 2: Fig. 6

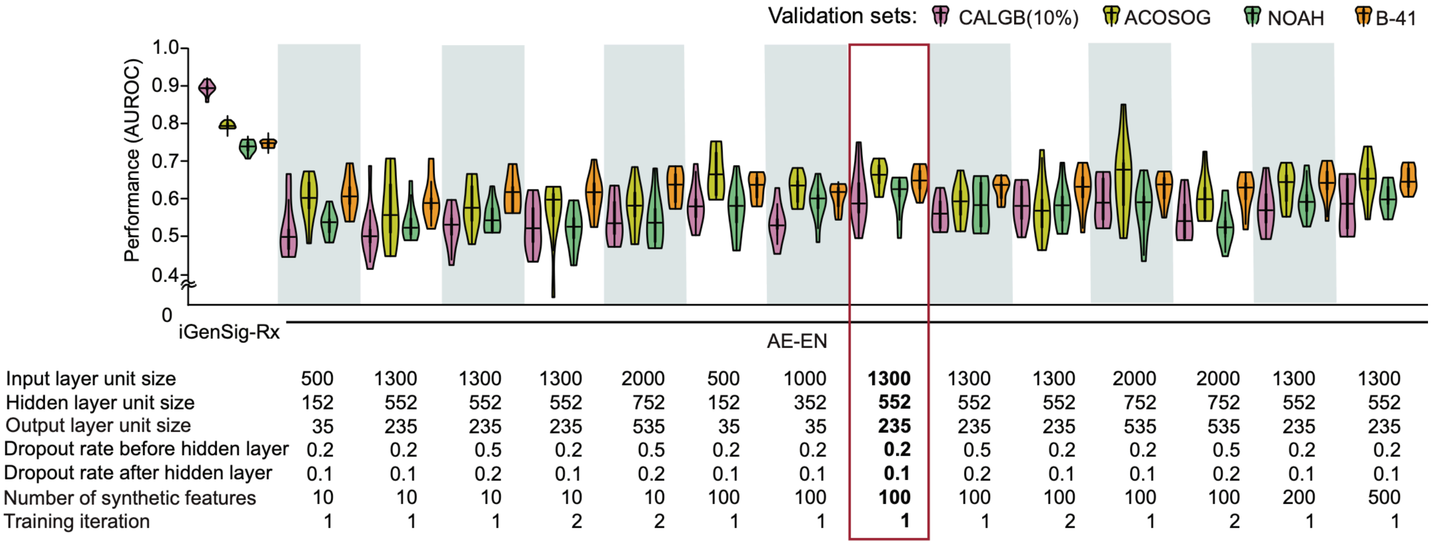

Input layer unit size	500	1300	1300	1300	2000	500	1000	**1300**	1300	1300	2000	2000	1300	1300
Hidden layer unit size	152	552	552	552	752	152	352	**552**	552	552	752	752	552	552
Output layer unit size	35	235	235	235	535	35	35	**235**	235	235	535	535	235	235
Dropout rate before hidden layer	0.2	0.2	0.5	0.2	0.5	0.2	0.2	**0.2**	0.5	0.2	0.2	0.5	0.2	0.2
Dropout rate after hidden layer	0.1	0.1	0.2	0.1	0.2	0.1	0.1	**0.1**	0.2	0.1	0.1	0.2	0.1	0.1
Number of synthetic features	10	10	10	10	10	100	100	**100**	100	100	100	100	200	500
Training iteration	1	1	1	2	2	1	1	**1**	1	2	1	2	1	1

Additional file 2: Fig. 6. Fine-tuning Autoencoder models by adjusting the embedding layer unit size, dropout rate, number of synthetic features, and training iteration. We used autoencoder for dimension reduction on 10 permuted training sets and applied elastic net for prediction on the internal and external validation sets, CALGB (10%), ACOSOG, NOAH, and B-41 datasets. The first column in the violin plot is the iGenSig-Rx performance and the rest of the columns show the AE-EN performance in the various tuning parameters. The iGenSig-Rx showed the best performance with the highest mean of AUROC and the lowest variance of AUROC of 10 permutations. The 2nd column is the autoencoder parameters that we used in this study that show the highest AUROC in NOAH and B-41 out of the fine-tuning test results.

Additional file 2: Fig. 7

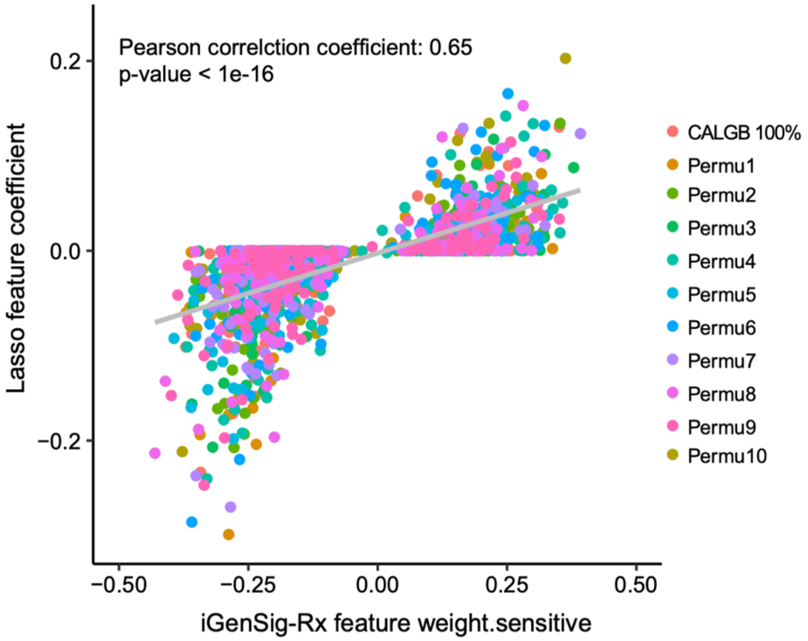

Additional file 2: Fig. 7. The correlation between iGenSig-Rx feature weights and lasso feature coefficient. The iGenSig-Rx sensitive feature weight and lasso coefficient were calculated in 100% CALGB 40610 and all 10 permuted 90% CALGB 40610 training sets. Features with lasso coefficient of zero were removed before calculating Pearson correlation coefficient.
